# Ethnoecology of the palm *Brahea dulcis* (Kunth) Mart. in central Mexico

**DOI:** 10.1186/1746-4269-11-1

**Published:** 2015-01-05

**Authors:** María T Pulido, Mayte Coronel-Ortega

**Affiliations:** Centro de Investigaciones Biológicas, Laboratorio de Etnobiología, Universidad Autónoma del Estado de Hidalgo, Carretera Pachuca-Tulancingo Km 4.5 s/n, Pachuca, Hidalgo, C.P. 42184 México

**Keywords:** Arid and semi-arid areas, Defoliation experiment, Disuse, Hñä hñü, Non-timber forest product, Population structure, Reserva de la Biósfera Barranca de Metztitlán, Sierra Madre Oriental, Sustainable use, Valle del Mezquital

## Abstract

**Background:**

There have been few studies on the sustainable use of non-timber forest products in arid and semi-arid zones. The palm *Brahea dulcis* has been one of the most important resources in semi-arid Mesoamerica, since pre-Hispanic times. Currently, some populations grow within protected natural areas, representing both a challenge and an opportunity for local development. This ethnoecological study of *B. dulcis* in central Mexico aimed to evaluate their uses, harvesting context, and potential for exploitation, in order to give practical advice on their best use and management.

**Methods:**

Ethnographic and ecological information was obtained in Barranca de Metztitlán Biosphere Reserve and Valle del Mezquital, Mexico. We studied the population structure and density; additionally, we evaluated the rate of leaf production, leaf renewal rate, percent survival of new leaves, the development of reproductive structures and performed a one-year defoliation experiment (involving a control and four treatments including a mix of semiannual and annual frequency of harvest and removal of two new leaves and/or two mature leaves).

**Results:**

Twenty uses of the palm were recorded in the study area. Religious/symbolic and handicraft uses are highlighted. The population density of this species was the highest reported for the genus (1244 ± 231.7 ind/ha). The leaf production rate was the highest reported for arborescent palms of the Americas (11.83 ± 0.036 leaves/individual/year). The sexual reproductive cycle was 2.3 years long. A one-year defoliation experiment did not show statistically significant differences. Recommendations include: 1) implement management focused on increasing the abundance and quality of this useful resource in Metztitlán; 2) employ a strategy of focusing on ethnicity and gender in promoting their exploitation; 3) learn from theoretical frameworks of other non timber forest product studies.

**Conclusions:**

We propose that *Brahea dulcis* is the palm with the highest potential for sustainable use in the arid and semi-arid zones of Mexico. The challenge to improving management includes simplifying the legal protection framework, promoting uses and developing a market strategy. Collaborations to share experiences with peasant farmers from Guerrero is recommended. We further recommend the development of a governmental strategy to enhance and reassess this important resource.

**Electronic supplementary material:**

The online version of this article (doi:10.1186/1746-4269-11-1) contains supplementary material, which is available to authorized users.

## Resumen

**Antecedentes:**

Existen pocos estudios sobre el uso de productos forestales no maderables en zonas áridas y semiáridas. La palma *Brahea dulcis* ha sido un recurso importantes en Mesoamérica desde tiempos prehispánicos. Actualmente, algunas de sus poblaciones están dentro de áreas naturales protegidas, lo que representa un reto y una oportunidad para el desarrollo local. El presente estudio etnoecológico de *Brahea dulcis* buscó evaluar sus usos, el contexto de su aprovechamiento, así como su potencial de explotación, con la meta de proporcionar recomendaciones para su mejor uso y manejo.

**Métodos:**

Se obtuvo información etnográfica y ecológica en la Reserva de la Biósfera Barranca de Metztitlán y partes del Valle del Mezquital, México. Nosotros estudiamos la estructura poblacional y densidad; además, evaluamos la tasa de producción de hojas, la tasa de renovación foliar, el porcentaje de sobrevivencia de las hojas nuevas, el desarrollo de estructuras reproductivas y realizamos un experimento de defoliación durante un año.

**Resultados:**

Se reportan 20 usos distintos, sobresaliendo el religioso/simbólico y el artesanal. La densidad poblacional de la especie es la más alta reportada para el género (1244 ± 231.7 ind/ha). La tasa promedio de producción de hojas fue la más alta reportada para las palmas arborescentes en América (11.83 ± 0.036 hojas/año). El ciclo reproductivo sexual tomó 2.3 años. Durante un año se aplicó un experimento de defoliación sin encontrar diferencias estadísticamente significativas entre éstos. Se recomienda: 1) implementar prácticas de manejo enfocadas en aumentar la abundancia y calidad de este recurso útil en la Reserva; 2) emplear una estrategia de promoción de su aprovechamiento basada en una visión étnica y de género; 3) aprender del marco teórico disponible en los estudios de no maderables.

Nosotros proponemos que *Brahea dulcis* es la palma con mayor potencial de uso sostenible en las zonas secas de México. El reto para lograr un mejor manejo incluye simplificar el marco legal, promover sus usos en contextos urbanos y rurales y desarrollar una estrategia de mercado. Se recomienda las colaboraciones para compartir experiencias con campesinos de Guerrero, así como desarrollar una estrategia gubernamental para realzar y revalorar este importante recurso.

## Background

The palm is the archetypical non-timber forest product (NTFP); since the dawn of humanity it has served many purposes [1]. There are numerous genera and species of palms typical of arid and semi-arid areas (A-SA), including *Brahea, Copernicia, Phoenix,* and *Washingtonia*. However, there are fewer studies of sustainable management of A-SA palms than of palms in humid areas [2]. In our opinion, it is very important that A-SA resources be studied more extensively for a variety of reasons, including: a) A-SA make up about 41% of the land area of the earth and these zones are increasing in area [3]; b) they are the predominant zones in some countries and regions; and c) some 33% of the world’s population lives in these ecosystems [3].

Much of Mexico’s land area is A-SA, where genera such as *Brahea* and *Washingtonia* evolved. 10 of 13 *Brahea* species are endemic to Mexico, as is *W. robusta*. Extensive areas of Mexico, located particularly in poor rural regions, are dominated by *Brahea dulcis,* where it clearly has great cultural, religious and economic importance currently and in the past [4–6]. For example, it is heavily traded in several major traditional markets, namely Tehuacán (Puebla), Sahuayo (Michoacán), and Tlapehuala and Chilapa (Guerrero) [7]. The use and commercial importance of this NTFP is so large that in the Chilapa market alone, which may be where the most of the species is sold, it is estimated that an average of 400 tonnes are sold per month (Cati Illsley, pers. comm., Sept. 2012).

*Brahea dulcis*, “palma sombrero” or “soyate”, has been one of the most important NTFP in the arid and semi-arid zones of Mesoamerica since pre-Hispanic times. Virtually all parts of the plant have been heavily used as NTFP. In pre-Hispanic times, tribute was paid to the Aztec empire with baskets and mats made of this species [6]. In Colonial Mexico, the palm hats made by the Franciscan friars became a major industry in the nineteenth and early twentieth century [4]: in 1877, 46,392 hats were produced in the state of Guerrero [8]. From ancient times, and still today, palm blades have been used to weave a basic piece of furnishing, the *petate*, a thin mat used as a floor covering and sleeping mat. The *petate* is part of the material culture of some indigenous groups (v.g. Nahuas).

Even today, the palm is used for religious offerings, and to make a variety of utilitarian items; humans have put almost every part of these palm plants to some use. The young leaves are used to make mats, hats, and brooms, while the older leaves are used in flower arrangements, and the dried leaves are used as fuel [4–6, 8, 9]. The roots are used to make dish scrubbers, and the flowers and fruits are edible [6].

Some of the populations of this useful species grow within protected natural areas (PNA), specifically biosphere reserves: Barranca de Metztitlán, Tehuacán-Cuicatlán, and Sierra Gorda. The inclusion of useful species such as *B. dulcis* in PNA represents both a challenge and an opportunity within and outside of PNA, where the sustainable use of NTFP could be a key contribution to local development. There are important local ethnobotanical studies of *B. dulcis*[5, 6, 8, 10], and some ecological [11] and genetic studies [12, 13], but currently it is not clear how their ecological features can help or impede their harvesting. Of the few studies of *B. dulcis* focusing on management, Illsley et al. [6] evaluated the impact of harvesting leaves, while [14, 15] developed management plans for certain areas of the state of Guerrero, based on population studies and particularly on knowledge from peasant farmers.

The aim of the present paper was to conduct an ethnoecological study of *B. dulcis* in the Barranca de Metztitlán biosphere reserves (RBBM in Spanish) and adjacent areas in Hidalgo state in central Mexico. The study aimed to answer the following questions: Which types of products are and have been produced currently and in the past? Which populations have the most potential for exploitation? What is the rate of production of new leaves and how does it vary through the year? What is the growth rate of the petioles? How long do the leaves last? How long does each stage of leaf development last (emergence, new, mature, senescent)? What frequency and intensity of harvesting young leaves will maximize production of new leaves? How long does sexual reproduction take from flower production to ripe fruit? It was hypothesized that this NTFP is used in a broad variety of ways, and that the rate of new leaf production per individual would be low due to the arid conditions, but that the potential harvest per unit area as a whole would be significant due to the high densities of populations in the RBBM. Meeting the study objectives enabled us to suggest better ways to manage and use this NTFP in the semiarid regions of central Mexico, ideas that can be applied to other regions as well.

## Methods

### The study species

*Brahea dulcis, B. nitida* André and *B. salvadorensis* H. Wendl. ex Becc. are the only species of the genus whose range extends beyond the borders of Mexico into Central America. In Mexico, *B. dulcis* is prevalent in the Balsas Basin, extending to southern Oaxaca, the high part of the Papaloapan Basin, and along the Sierra Madre Oriental as far as southern Tamaulipas [16]. The species grows most abundantly between 1200 and 2200 m.a.s.l. in the understory of oak and pine-oak forests, low deciduous forests, and in disturbed areas where it may form almost monospecific palm groves that are apparently secondary [16, 17].

This palm grows up to 8 m tall, with sometimes solitary, erect stems and frequently has caespitose growth; in adults, the crown has 10–15 flabellate leaves, petioles with armed margins, inflorescences of sessile flowers hanging in modified racemes, and monocarpic, monosperm drupe fruits [18]. The palms are hermaphroditic plants with bisexual flowers [17]. They reproduce by both sexual and asexual means [6], as the suckers are fire-resistant [19]. They interact with ants and bees [9], which may serve as pollinators. The fruits are dispersed by birds, small mammals, coyotes, and humans. There is evidence that *B. dulcis* can hybridize with *B. nitida* when the two species grow sympatrically [13].

According to [4, 6] there are two types of *B. dulcis* palmars: a) the low-growing or “*manchonera*” palms about 1.5 m tall, which most often reproduce asexually, increasing the density of ramets; and b) the tall “*soyacahuitera*” palms six or more meters tall, in which sexual reproduction is more common. These two different morphologies are the result of management practices employed by the local people (intensive leaf harvesting, burning, and grazing), which keeps the palm trees short; moreover continual leaf harvesting increases plant population density [6]. A *manchonera* can grow to be a *soyacahuitera* under human management.

### Study area

Our study area was located within the A-SA regions of Hidalgo state in central Mexico, particularly the Barranca de Metztitlán Biosphere Reserve (RBBM) and adjacent areas of Valle del Mezquital. The RBBM is a 2,090,512-hectare PNA characterized by a high richness of cactus species, especially endemic Mexican cacti. The RBBM includes tropical arid scrub, tropical deciduous forest, submontane scrub, pine forest, pasture, and riparian woodland ecosytems [20].

**Figure 1:**
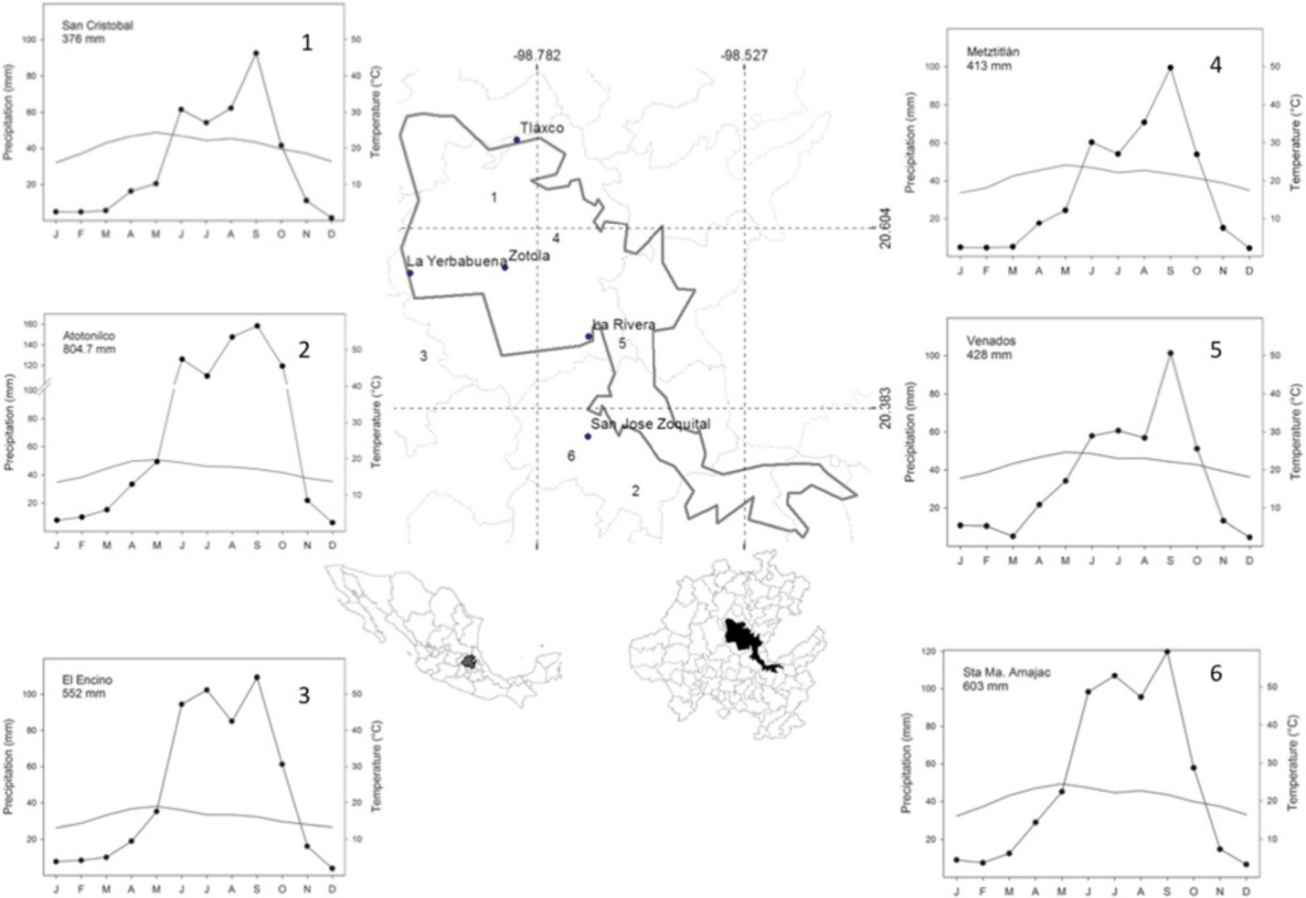
***B. dulcis*** **study sites and climate regions in the RBBM (polygon).**

The RBBM is located in the Huasteca Karst subprovince of the Sierra Madre Oriental province, where regional tectonic movements and erosion caused by the Amajac and Metztitlán Rivers carved deep canyons. The climate is generally hot dry and semi-dry due to the rain shadow effect of the Sierra Madre Oriental. Average annual precipitation is 500 mm, mostly in summer and less in winter. The rainy season lasts from June to October, but can be shorter in some parts of the region (Figure 1). The average annual temperature ranges from 18 to 22°C [20]. In the Valle del Mezquital the towns of Naxthe, Taxhie and Ixmiquilpan were studied. The predominant local culture in this semi-arid region is hñä hñü.

### Interviews

In March 2008, RBBM staff were interviewed about their opinions on the general status of the palm. They informed us that Tlaxco is currently the main local palm handcraft town. We then visited Tlaxco and Metztitlán for five days during Holy Week to conduct open interviews. Although at the beginning the people were somewhat reserved, all the respondents consented to be interviewed, especially when they learned that we were interested in talking about the palm and that we were from the university.

We asked for general ethnographic information including: 1) the home towns of harvesters and handcrafters currently and in the past; 2) the handcrafters’ opinion about the main problems and challenges relating to the use of this NTFP; 3) understanding the local commercial network; 4) finding out the types of palm products produced in the region. In this first exploratory trip we conducted open interviews with some twelve handcrafters in Tlaxco (all women, because there were no male handcrafters in the town). Additionally, we spoke with the main middleman in Tlaxco, to find out where he goes to sell the products and how they are marketed. Finally, we also spoke with one handcrafter and seller of palm goods in Metztitlán to learn their perspective. After two weeks, we returned to interview four sellers at the Ixmiquilpan market, to ask about where they found the handcrafters whose work they sold.

Between August 2008 and April 2009 we conducted 25 semi-structured interviews and carried out participant observation in Naxthé (2), Taxhie (2, in Alfajayucan municipality) and Tlaxco (21 in Metztitlán municipality). These places were chosen based on information obtained from sellers in Ixmiquilpan. At the beginning, the research was explained to the informants. Interviews were conducted with handcrafters to find out: 1) the type of products that they can make; 2) how long it takes them to weave each type of product; and 3) how many palm blades they require for each product. By means of participant observation, the second author worked closely with the handcrafters and learned how to make the crafted articles. Before the research was conducted, we asked permits from the authorities of the Biosphere Reserve and from Communal authorities ("Delegado"). Additionally, we explained the purpose of our study to each person interviewed and asked for his/her verbal consent.

### Potential for exploitation of palm populations

In June, 2008, we made a reconnaissance visit, guided by RBBM staff. They were interested in evaluating which populations have the greatest potential for exploitation, and suggested five sites for study: La Rivera, Tlaxco, La Yerbabuena, Zotola (Metztitlán municipality) and San José Zoquital (Atotonilco El Grande municipality). Five palm populations were of the *manchonera* type, and are now harvested almost exclusively for Palm Sunday, and the dry leaves for fuel. Each population’s potential for exploitation was estimated by assessing its density and population structure. The distance between sites ranges between 4.3 to 50 Km (Figure 1). The general characteristics of the sites are listed in Table 1.

**Table 1:** **Characteristics of** ***Brahea dulcis*** populations studied in the RBBM (Metztitlán and *Atotonilco El Grande municipalities)

Population	Localization	Elevation (m)	Harvest intensity	Human group
La Rivera	20° 28.241´N by 98° 43.105´W	1964	Medium	Mestizo
Tlaxco	20° 42.782´N by 98° 48.386´W	1902	null	Mestizo
La Yerbabuena	20° 32.921´N by 98° 56.248´W	1848	High	Hñä hñü
Zotola	20° 33.311´N by 98° 49.239´W	1907	Low	Hñä hñü
San José Zoquital or Atotonilco*	20° 20.914 N by 98° 43.154´W	2065	Low	Mestizo

At each site, a sampling area of 0.1 ha was chosen, made up of ten 10 × 10 m randomly selected quadrants. The size of the quadrants was chosen because we observed many individuals generally distributed in clusters. The total number of *B. dulcis* individuals in each quadrant was counted, the heights of the stipes were measured, and they were categorized. If two palms close together were joined at the root, it was counted as one individual. Height was measured from the ground to the apical meristem. In individuals with prostrate or curved stems, the convex side of the stipe was measured. To characterize the population structure, individuals were subdivided into five classes based on the recommendations of Balslev et al. [21] for studying palms, although we made some changes; our classes were defined as: 1) seedlings (S): lanceolate leaves, stems of some centimeters, sucker absent; 2) saplings (I): leaves partially divided, similar size to seedlings, sucker absent; 3) juveniles (J): flabellate leaves, stipe to one meter, can present suckers; 4) subadult (SA): flabellate leaves, stipe generally at least one meter, can present suckers; 5) adults (A): flabellate leaves, stems generally at least 1.5 m, can present suckers, reproductive.

The population density by site was calculated for the total number of individuals and for each size category. For each individual, we also recorded whether the palm had harvestable leaves; that is, leaves large enough to be useful. Total density was calculated, and the population structure was graphed, distinguishing between individuals that were useful for supplying handcraft materials and those that were not.

### Rate of leaf production, leaf renewal rate and percent survival of new leaves

Since harvesting the leaves can influence the production of new leaves, leaf production was measured every two weeks at San José Zoquital, the site where leaves are harvested the least by the local residents (Table 1). Every two weeks, from July 2008 to July 2009, 42 individuals were observed. These palms were in the useful range of 0.6 to 1.6 m tall; harvesters can reach their leaves from the ground. In order for the sample to be statistically independent, palms grouped in patches were excluded, since it is known that individuals located close to each other may be genetically identical [13].

At the first sampling, each individual was labeled and located geographically, its total height was measured and the number of green leaves, inflorescences and infructescences counted. Following this, at each fortnightly sampling, new leaves were marked (with paint on the petiole), using a different color each time. Leaves, inflorescences and infructescences were each marked individually in order to identify and record their stage of development.

The following data were recorded every two weeks for each of the 42 selected individuals throughout the one-year sampling period: a) the number of new leaves per individual – the average yielded information on leaf production rate per individual and variation over time; b) the length of the new leaf petiole, to evaluate the average rate of growth (this was only measured in 179 leaves on 15 individuals); c) the first new leaf produced by each individual during the observation period – these were marked and followed in order to measure the average annual leaf survival rate.

To measure leaf turnover, all the leaves of each individual were classified as new, green or dry. A new leaf (NL) was defined as one that is budding out from the apical meristem, with petiole visible and leaf blade at most partially opened (the part closest to the petiole no more than 15 cm wide). A green leaf (GL) was a leaf with a blade more than 15 cm wide and bright green in color. A dry leaf (DL) was brown in color and no longer borne erect.

### Defoliation experiment

Due to differences in harvest regime between populations, we decided to conduct defoliation experiment at the La Yerbabuena site, which is the most heavily harvested site (Table 1), so that the resulting potential management recommendations would be applicable to a realistic harvesting scenario. The defoliation experiment was conducted for a period of one year (July 2008 to July 2009) on 100 individuals, to which a total of four different treatments and a control were applied. A random block experimental design was applied, using 20 blocks throughout the site. Each block consisted of five individuals, one randomly assigned to each treatment and the control.

The treatments involved a combination of different harvesting frequencies (semiannual or annual) and intensities (two new leaves and/or two mature leaves) (Table 2). The experimental treatment was determined based on what had been done in other studies with mature leaves. Although in this case the useful resource is the new leaves, in some treatments (T3 and T5, Table 2) we decided to harvest both new and mature leaves since harvesting mature leaves can potentially stimulate physiological responses in the plant that can increase the production of new leaves.

**Table 2:** **Defoliation treatments applied to 100** ***Brahea dulcis*** **individuals for one year at La Yerbabuena (Mexico)**

	Control	T2	T3	T4	T5
New leaves harvested (#)	0	2	2	2	2
Mature leaves harvested (#)	0	0	2	0	2
Harvesting frequency	never	semiannual	semiannual	annual	annual

The 100 individuals in the experiment had heights ranging from 0.6 to 1.6 m high, this being the range of plants that are usually harvested. Each individual was labeled and georeferenced (GPS), and the newest, second-newest and third-newest leaves were marked with a dab of colored paint on the petiole in order to differentiate between treatments. Data recorded for each plant at the beginning and end of the experiment were total plant height (ground to base of the petiole of the youngest leaf), total number of leaves, and the number of inflorescences and green and dry infructescences. The numbers of new and green leaves were recorded before the experimental treatments were applied. In January 2009, the numbers of leaves produced over the previous six months of the experimental period were counted for individuals in the T2 and T4 groups, and leaves were then harvested according to the respective treatment.

### Development of reproductive structures

**Figure 2:**
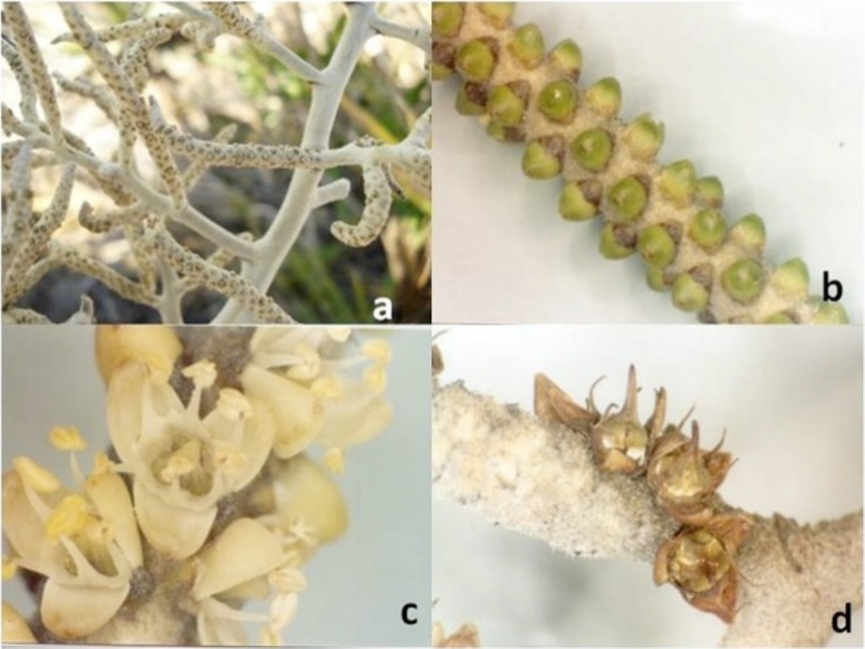
**Stages of development of** ***B. dulcis*** **inflorescences: a = pubescent, b = bud, c = flower, d = dry flower.**

The time from emergence of inflorescences to mature fruit production was recorded on the 42 study individuals. Eighteen of them had inflorescences or infructescences during the study period. The reproductive structures were marked as described in the section “Rate of leaf production, leaf renewal rate and percent survival of new leaves” and classified into developmental stages.The inflorescences were classified into five stages: emerging, pubescent, bud, flower and dry flower (Figure 2). Emerging inflorescences did not yet show rachillae, and were dark red. Pubescent inflorescences had smooth-textured, pearlescent cream-colored rachillae. The bud stage showed immature green petals. The flower stage corresponded to full anthesis, with the colorful whorls visible. In the dry flower stage, the floral whorls were dark brown. The infructescences were classified into four categories according to the dominant color of the fruit; green, yellow, black, or dry (Figure 2).

These data were used to estimate the total duration of flower and fruit production, and the duration of each stage. It should be noted that the size of the sample was different for each stage, as it depended on flower and fruit production in the sample of plants.

### Data analysis

The ethnobotanical information was summarized in a table. The population structure was compared between localities using the chi-square statistic and *a posteriori* adjusted residual analysis described by Habermann [22]. The potential for use by site was evaluated on the basis of density of useful individuals.

The leaf production rate per individual (mean ± 1 s.e.) was calculated for each two-week period and averaged for the 42 individuals to give the fortnightly leaf production rate per individual and its variation across fortnightly periods. In addition, the rate (by month) was related to average monthly rainfall of the current and previous month [23], available for the period 1991–2006 using a simple linear regression model.

To explore which variables could explain leaf production per plant, we used a generalized linear model (GLM) that had the leaf production rate of each individual as the response variable, and plant height, height squared, the number of suckers and number of reproductive structures as explanatory variables. In these models, the parameters and random variables are necessarily linear, while the mathematical variables may not be [24]. Since the response variable was leaf counts, we used a Poisson distribution. A chi-square function was used to evaluate the statistical significance of the variables. The GLIM statistical package (Numerical Algorithms Group, UK) was used.

Average growth rate of the leaf petioles per individual was calculated by averaging the growth values of each petiole (for example, the average growth in the first two-week period of the 6 to 17 leaves produced by each plant) even though these were produced in different periods. The average leaf growth rate for the 15 individuals observed was also averaged and graphed. The percentage survival of the first leaf (FL) produced by each of the 42 palm plants was calculated (no. of green FL/42 × 100). The length of the reproductive cycle was calculated by adding together the times from all nine stages.

## Results

### Ethnobotany

According to our ethnographic information, people have been harvesting only the leaves for at least eight decades in the region. They take them to the markets, or directly to the craftspeople, but there are no useful palms in the towns where the craftspeople live (Tlaxco, Naxthé, Taxhie). Ixmiquilpan, Actopan and Atotonilco are the main palm markets in the region; Metztitlán is a smaller, seasonal market which is most active in Holy Week. Ixmiquilpan is the most important market for trade of food, fibers such as ixtle, and palm, and has been so since pre-Hispanic times. There is a considerable geographic division between craftspeople and harvesters. As a result, transporting the material is an essential part of the process, in the past on donkeys and now in trucks.

**Figure 3:**
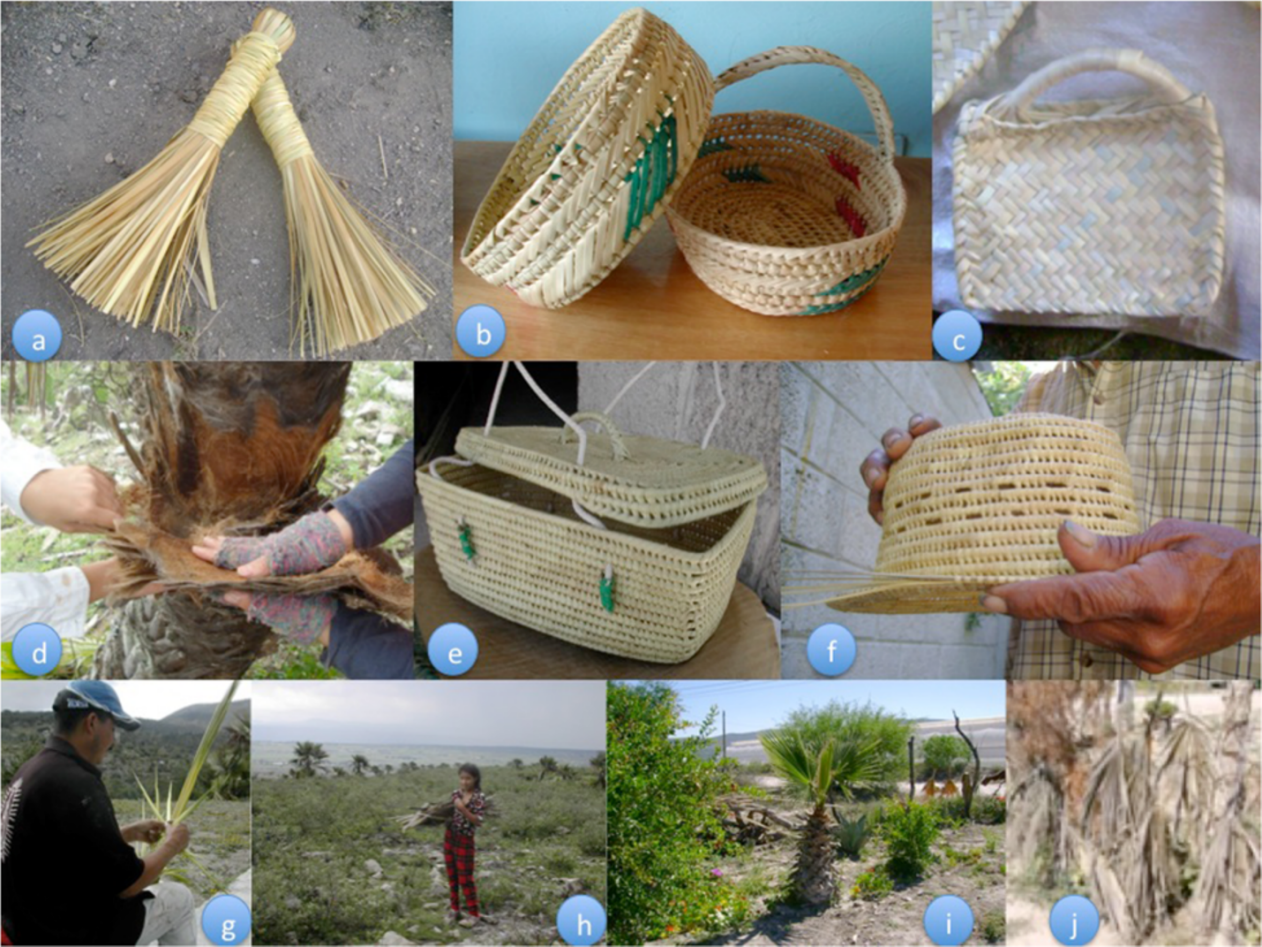
**Uses of** ***Brahea dulcis*** **in Hidalgo State: a) brooms, b) baskets, c) fans, d) foliar bracts, e) bags, f) hats, g) “** ***ramo*** **” or branch used on Palm Sunday, h) fuel, i) ornamental, j) fence.**

In Tlaxco, Naxthé, Taxhié, the most common handcrafted palm products are petates (18 of 25), followed by brooms (12/25), fans (4/25), hats (4/25) and miniature implements (3/25). Petates require 15 to 40 leaves and one to seven days to make but are not the most profitable product; a hat takes the same time to make but sells for a higher price (Table 3). Twenty different types of products are crafted in the region as a whole (Table 4); the most common uses for the palm were found to be utilitarian and religious/symbolic objects. Utilitarian items include hats, bags, and brooms (Table 4, Figure 3). They also used to include “*capotes*” or raincapes that currently are in disuse (replaced by plastic); photographs of *capotes* were collected in the ethnographic work of [10, pp 65].

**Table 3:** **Number of leaves, time required and selling price of the main products of** ***Brahea dulcis*** **made in the towns of Tlaxco, Naxthé and Taxhie**

Product	Leaves required (#)	Time required	Selling price (Mexican pesos)
Hat*	N.A.	5-7 days	70 - 90
Fan	0.5	1 hour	3
Mat	15- 40	1-7 days	20 - 40
Broom	N. A. Leaf scraps left over from making *petates*	30 minutes	3 - 5

*Leaves are boiled before weaving. N.A. = not available.

**Table 4:** **Uses of** ***Brahea dulcis*** **in the RBBM and adjacent areas**

Part	Product	Uses/Description	Place where made or sold
Entire plant		Ornamental: decorate houses and gardens	San José Zoquital
New leaves	Strips	Raw material: strips used to form woven palm hats or other objects	Ixmiquilpan market
	Mats	Handcraft: sleeping mat.	Tlaxco, Naxthé Taxhie
	Fans	Handcraft: tool to fan fire in the stove	Tlaxco, Naxthé Taxhie and Ixmiquilpan market
	Broom	Handcraft: to sweep	Ixmiquilpan
	Whisks	Handcraft: to clean ashes from the stove.	Ixmiquilpan
	Tortilla baskets	Handcraft: container to hold tortillas.	Ixmiquilpan, Naxthé, Taxhie and Tlaxco
	Bags	Handcraft	Tlaxco, Naxthe and Ixmiquilpan market
	Hats	Handcraft	Tlaxco, Naxthe Taxhie
	Miniature implements	Handcraft and commercial: miniature household implements used as brooches, earrings	Ixmiquilpan, Naxthe, Taxhie and Tlaxco
	Branch	Religious: branch used in Palm Sunday ceremonies	Tlaxco, Naxthé and Ixmiquilpan market
	Burial headpiece	Religious: burial headpiece	Tlaxco, Naxthé Taxhie
	Leaf strips	Commercial: for tying vegetables	Ixmiquilpan market
	Leaves	Cooking: fresh leaves are used to wrap *zacahuil* (traditional Huastecan food)	Huejutla de Reyes
	cloaks or raincapes (*capote*)*	Handcraft: garment for rain protection	Juárez Hidalgo
Green leaves	Decoration	Ornamental: for flower arrangements	Ixmiquilpan,
	Fences	Domestic: to delimit land.	Naxthé
Dry leaves	Combustible	Fuel	San José Zoquital
Fruit	Food	Alimentary: human food; the fruit is called *capulín*	Atotonilco
Foliar bracts	Pads	pads for donkeys	Ixmiquilpan market

*Disuse.

Religious uses include the branch or “*ramo*” used on Palm Sunday in many towns (Table 4) and the burial headpiece reported for the first time. In Taxhie, the dead are buried with a woven palm leaf headpiece shaped like a simple crown bearing a small cross. A village tradition says that once a man who died in Taxhie met St. Peter and was asked why he arrived with his head uncovered. St. Peter explained that this is a requirement for entering heaven. The man was told that he could return to earth and tell people that they had to be buried with a headpiece. The role of protection attributed to the palm also includes the power of calming storms when the leaves are placed or burned behind the main door of a house (in Hidalgo and Mixteca Oaxaqueña).

Many of these uses require leaves. There are two ways to process the new leaf to make the final product: either the new leaf is cut, boiled, dried in the sun and woven, or it is only cut and woven. The results are different. Boiled and dried, the leaf is more flexible and easier to weave, and the final product is lighter in color. In the latter case, the leaf is stiffer, darker in color and more readily torn or broken. The majority of products are made with new leaves (not expanded yet). Dead leaves are currently used as fuel and until a few decades ago, were used for thatching traditional roofs of houses.

### Variation among populations in potential for exploitation of palm

**Figure 4:**
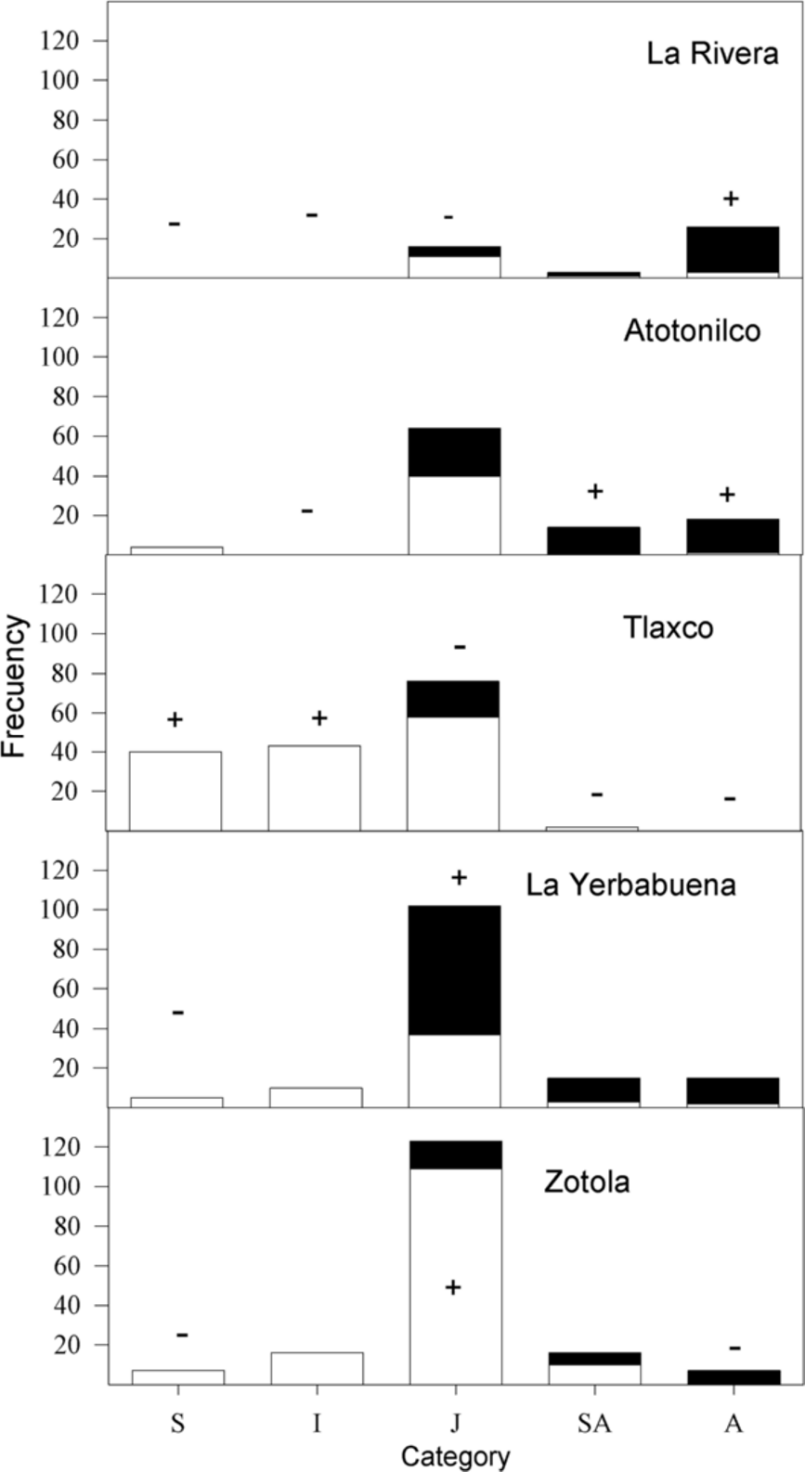
**Population structure of** ***Brahea dulcis*** **at five sites in the RBBM, in sampling areas of 0.1 ha per site.** The frequencies of individuals with harvestable leaves are shown in black and with non-harvestable leaves in white.

The differences in population structures of *B. dulcis* between the five study sites were statistically significant (χ^2^ = 269.3, P <0.05, df = 16; Figure 4). Populations consistently tended to have low seedling densities and a very low density of individuals > 2.01 m (Figure 4), but the population as a whole showed significant densities in the RBBM (1244 ± 231. 7 ind/ha), with a strong variation between sites (450 to 1690 ind/ha, Table 5). In descending order of total density, the sites are Zotola > Tlaxco > La Yerbabuena > Atotonilco > La Rivera (Table 5). Ordered by density of useful individuals, however, the sites are Yerbabuena > Atotonilco > La Rivera > Zotola > Tlaxco. The total density of the densest population (Zotola) was 3.76 times that of the least dense population (La Rivera). The density of useful individuals (defined as those bearing leaves useful to craftsperson) differed even more across populations. By this measure, La Yerbabuena was 5.1 times denser than Tlaxco (Table 5).

**Table 5:** **Comparison of total density and density of useful individuals of** ***B. dulcis*** **counted in 0.1 ha (10 plots of 10 × 10 m per site) at five sites in the RBBM**

	Density	Density of useful individuals (#/0.1 ha)	Useful individuals (%)
La Yerbabuena	147	91	62
Atotonilco	100	55	55
La Rivera	45	30	67
Zotola	169	27	16
Tlaxco	161	18	11
AVERAGE	124.4	44.2	42.1
S.E.	23.1	13.2	11.8

### Rate of leaf production, leaf growth rate and percent survival of new leaves

**Figure 5:**
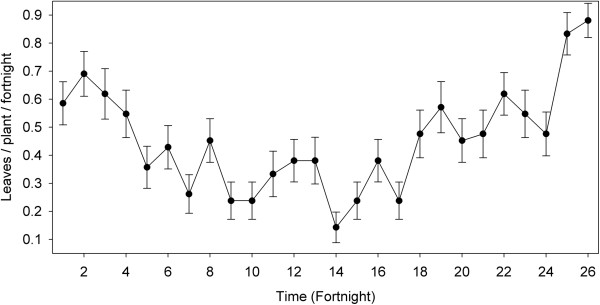
**Fortnightly leaf production per individual (mean ± 1 s.e.) of** ***Brahea dulcis*** **in the RBBM.**

One palm produced on average (±1 s.e.) 11.83 ± 0.036 leaves per year, with a notable variation among individuals (ranging from 5 to 19). Leaf production was positively correlated with plant height (R^2^ = 0.3805, P <0.05). Trunk height, the only significant variable in the GLM, explains 30% of the total variation in leaf production. In the rainy season (June to October) leaf production was 0.60 ± 0.05 leaves per individual per fortnight, and in the dry season (November to May) it was 0.35 ± 0.03 leaves per individual per fortnight. The extremes of leaf productivity ranged from 0.88 new leaves per individual per fortnight (July 2009) to 0.14 new leaves per individual per fortnight (February 2009) (Figure 5). Leaf productivity showed a statistically significant relationship with average monthly rainfall in the same month (R^2^ = 0.3328, P = 0.050) and the previous month (R^2^ = 0.5105, P = 0.013).

**Figure 6:**
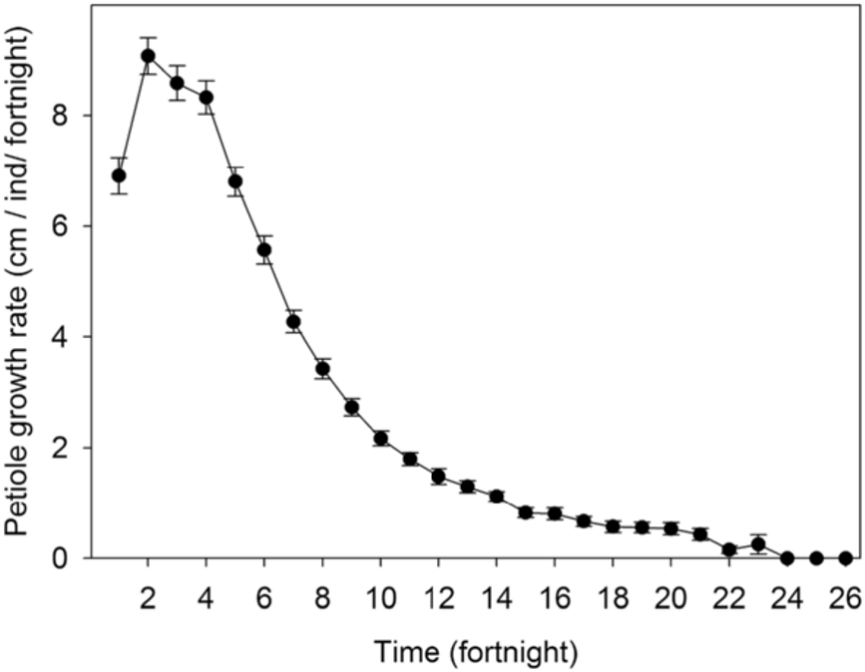
**Fortnightly growth of petiole in** ***B. dulcis*** **.**

**Figure 7:**
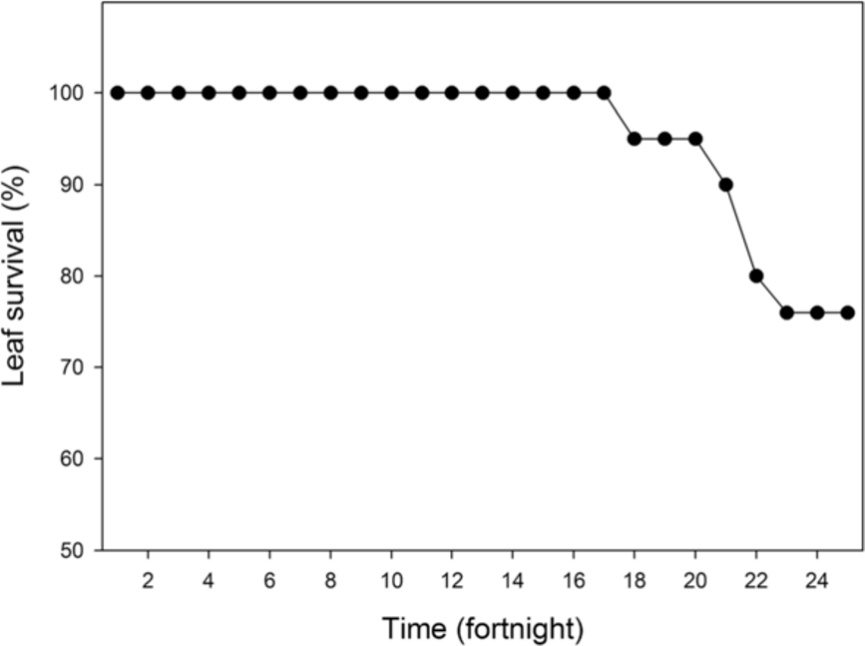
**Percent survival of** ***Brahea dulcis*** **leaves in the RBBM.**

The length of the leaf life cycle was 24 fortnights, during which time the leaf grew from the new to the green stage and in some cases to the dry leaf stage. The petiole showed its highest growth rate (9 cm) during the second fortnight and then began to decrease with a negative geometric distribution (Figure 6). A leaf lasted on the plant an average of 20.18 fortnights, and the GL stage was twice as long as NL and DL (Table 6).The 21 leaves marked for the study of leaf survival had an annual survival rate of 76% (Figure 7). The first leaf deaths occurred in the 17th fortnight, and continued gradually decreasing.

**Table 6:** **Durations of leaf stages in** ***B. dulcis***

Leaf stage	Duration (fortnight ±1 s.e.)	Range (fortnight)
New leaf	4.11 ± 0.17	1.9 a 6.4
Green leaf	10.59 ± 0.30	7.1 a 16.9
Dry leaf	5.48 ± 0.42	2.0 a 15.0
TOTAL	20.18	

The GLM showed no statistical differences between leaf productivity by experimental treatment (X^2^ = 2.53; g.l = 4; P = 0.6387). The leaf production rates per treatment were: control 8.60 ± 0.38, T2 9.0 ± 0.4, T3 9.7 ± 0.58, T4 8.15 ± 0.5 and T5 8.65 ± 0.5 leaves/plant/year.

### Reproductive structure development stages and duration

Throughout the year, it was always possible to find at least one individual in nearly every stage of inflorescence and infructescence; in general the reproductive cycle is not associated with particular times of the year, and there is little synchronization among individuals (Table 7). There was however, synchronization within individuals; their reproductive structures moved through the stages almost simultaneously (Additional file 1). Emergence of inflorescence was the only stage restricted to a particular season of the year: emergence was only observed from April to June, as the season changed from dry to the beginning of the rainy season. However, the duration of a single stage often varied greatly between different individuals, especially the stages of flowering, dry flower, yellow fruit and black fruit (Tables 7 and Additional file 1). For example, the flower stage varied from 3 to 10 fortnights among different individuals.

**Table 7:** **Summary of reproductive stages of 18** ***B. dulcis*** **individuals observed for one year in the RBBM**

Stage	Duration in fortnights ± 1 s.e. (range)	N complete	N underestimated	average structures/individual/fortnight	Months
Emerging	3 ± 0	4	4	2 - 4	April–June
Pubescent	14 *	1	4	3 - 4	July–January (n = 1) and June–July (n = 4)
Bud	10.62 ± 1.45 * (5 – 17)	1	9	1 - 2.5	July–April
Flower	6.65 ± 1.47 (3 – 10)	6	0	1 - 2.5	Sept–July (main Nov–April)
Dry flower	2.67 ± 0.56 (1 – 5)	6	0	1 - 3	Sept–July (main Feb–May)
Total inflorescence	36.9				
Green	7.20 ± 1.48 (1 to 14.8)	1	10	2 - 4	July–Feb (n = 9) and May–July (n = 1)
Yellow	4.69 ± 0.94 (1–9)	9	0	2.0 - 5.5	August–April (main Sept–Dec)
Black	6.37 ± 1.06 (4–14)	9	0	2.0 - 7.0	Nov–July
Dry	--			2.8 - 4.57	year-round
Total infructes-cence	18.26				
Duration	55.2				

N complete = number of reproductive structures where the entire stage was observed. N underestimated = number of reproductive structures where a partial stage was observed (had already started development before observation period, or stage not completed at end of observation period). When N is two or more, the standard error (s.e) is provided. Details in Additional file 1.

**Table 8:** **Comparison of leaf production rate in arborescent palms of the Americas (sorted by annual precipitation)**

Species	Vegetation type	Annual precipitation (mm)	Years of study (#)	Leaf production rate (leaves/in-dividual/year)	Source
*Brahea dulcis*	Palm forests	500	1.0	11.83	This study
Thrinax radiata*	Dry tropical forest	1100	1.5	2.85**	32
*Euterpe edulis*	Subtropical moist forest	1371	1.0	2.21	30
*Oenocarpus bataua*	Premontane forest	1995	9.4	0.72 to 3.36	41
Ceroxylon echinulatum*	Cloud forest	2000 to 4000	2.0	2.05 ± 0.69	42
*Phytelephas aequatorialis*	Moist and very moist tropical forest	2300	1.0	2 or 3 (generally)***	43
*Astrocaryum mexicanum*	Tropical rain forest	4600	6	1.34 seedlings to 2.55 for immature and mature plants	44
*Prestoea montana*	Subtropical rain forest	4661	36	4 ± 0.14	45

*Control plants. **2.85 is the sum of semester one (1.35 leaves) + semester two (1.5 leaves, see Figure 1 in the original paper). ***see Figure 6 in original paper.

Based on the data (Tables 7 and Additional file 1), which is limited for some stages, the best estimate for the average length of inflorescence is 36.9 fortnights, and for infructescence (except the dry stage) 18.26 fortnights. The total reproductive cycle from the emergence of the inflorescence to mature fruit production takes at least 55.2 fortnights; that is, 2.3 years.

## Discussion

### *Ethnobotany: use and disuse of*B. dulcis

Although some significant ethnobotanical research of this species has been undertaken, such as the work of [5] in Huizltepec (Guerrero), [10] in Michoacán and [6, 8] in La Montaña de Guerrero, as well as the present study, no thorough ethnobotanical studies at the national scale have yet been published. Our study reports new uses including miniature implements, the burial headpiece, leaf strips for tying bundles of vegetables, and leaves to wrap *zacahuiles* (a type of tamal).

While the species is and has been extensively used currently and in the past, certain uses have greatly decreased or discontinued. These include the use of the leaves to make *capotes* (rain capes), *petates* (sleeping mats), and the substitution of leaves used for thatching and handcrafts by synthetic materials (it was once common in the study region for house roofs to be thatched with the leaves of this palm). The substitution phenomena is common to many NTFP, caused by resource scarcity and the lower prices of some synthetic materials [25]. In the RBBM, substitution and decreased use of *B. dulcis* is due to difficulty in obtaining the raw material (whether legally or illegally) and probably to emigration, cultural change and/or low prices of palm products. The problem of obtaining the raw material is due to the difficulty of meeting the requirements of current regulations. For example, when the RBBM was established, the people of the hñä hñü village of El Palmar, which owes its name to the high abundance of *B. dulcis*, submitted an official letter complaining of legal impediments to the use of the palm, which have not been resolved to date [26].

In spite of the trend of decreased use, maintaining use is very important because it not only produces useful objects, but is also part of cultural identity, as well promoting commerce. Additionally, objects made of NTFP help the carbon sink, and offer a better alternative than plastic to fight climate change.

### Density and population structure

The density of *B. dulcis* is an order of magnitude greater than that reported for other species of *Brahea*, corroborating its value as a NTFP and supporting the hypothesis that the potential for its exploitation per unit area in the RBBM is high. Extrapolating our results, *B. dulcis* showed an average density of 1244 ± 231.7 ind/ha (Table 5) compared to 278 ± 66.9 ind/ha (unpublished data) recorded for *B. armata*, a species endemic to the Baja California Peninsula, at six desert canyon sites. For *B. aculeata*, endemic to Sonora, the average density was 121.7 ± 36.3 ind/ha in tropical deciduous forest [27]; the variation between species is extensive.

The densities of *B. dulcis* found in the RBBM (1244 ± 231.7 ind./ha) are very close to those obtained by [6] for *B. dulcis* at 18 sites in the Chilapa region (Guerrero, Mexico), where a density of 1157.9 ± 174.6 genets/ha (calculated from 6, Table 2) was reported, ranging from 350 to 3233 genets/ha across sites. Clustered palms were counted as one genetic individual or genet in both studies, making comparison possible.

Current density and population structure of *B. dulcis* in the RBBM vary significantly between sites, meaning that the potential for exploitation varies greatly from one locality to another. Thus, while Zotola has the highest density, many individuals are juveniles with prostrate stems and are therefore not suitable for harvest. The area is heavily eroded and grazed (the latter particularly in the past). Tlaxco had the next-densest population, but it was dominated by juveniles, which are not useful, and by seedlings and saplings, which are not yet useful. Curiously Tlaxco had a scarcity of adults, but seeds appear to be originated by individuals present in numerous ravines which were not sampled.

La Yerbabuena, Atotonilco and La Rivera had the lowest density of total individuals, but the highest density of useful individuals. Adults made up a significantly higher proportion at La Rivera and Atotonilco, contributing to management potential. Additionally, the three populations share the characteristic that leaves are harvested at least twice per year, contributing to higher levels of use and management. More precise measurement of the intensity of management of *B. dulcis* could be an important aspect of future research, by means of appropriate indexes such as that of [28].

Ramírez-Rodríguez et al. [13], building on the system described and studied by Illsley et al. [6], recently found that management increased genetic diversity (heterodicity), increased the density of genets and ramets in the population, and increased the number of genotypes in *B. dulcis*. Thus the highest density was observed at the La Yerbabuena site, followed by Atotonilco, which could be in part a result of more intensive management at these places compared with the others.

### Leaf longevity and production rate

Our results are consistent with leaf longevity reported for the species in the state of Guerrero, where [15] notes that it varies from 8 to 14 months, and is directly proportional to the height of the individual. Small farmers in Guerrero know that the leaves take eight months to complete their life cycle [15], similar to our data. Illsley et al. [6] report an annual rate of 20 leaves per year, higher than our results; these differences may be due primarily to the fact that these authors selected tall palms, while the present study included relatively short individuals.

When these results are compared to those recorded for other palms, it can be seen that *B. dulcis* annual leaf production is the highest reported for arborescent palms of the Americas (Table 8), while leaf longevity is low. This suggests that the strategy of *B. dulcis* is to produce many leaves, but with each leaf having a relatively short life. This makes it ideal from the point of view of sustainable NTFP use, since although removing leaves for handicraft uses removes photosynthetic production for an average of 20.18 fortnights, which has an impact on the energy balance of the plant, at the same time the characteristics of the palm’s life cycle – high leaf productivity and short leaf life cycles – mean that humans have less effect on the plant while they are taking advantage of its life cycle.

The findings for *B. dulcis* are consistent with the trends described by [29] for palms of the world, in which he found a positive relationship between leaf production rate and the number of leaves in the crown. He also reported a negative correlation between the number of leaves produced per year and leaf longevity. This suggests, furthermore, that the more dry and seasonal the climate, the more leaves there tend to be in the crowns of the palms. He also found that the number of leaves per tree is higher in dry than in wet habitats. Petiole growth pattern was similar to that reported for *Euterpe edulis*[30].

### Optimal harvest

This study is the first to have the stated goal of optimizing new leaf production. Our results are consistent with the results reported by [15], who compared leaf production between harvested and unharvested plots during a two-year period. This failure of the plant to show a response to the experimental treatments we applied is largely due to the fact that few leaves were harvested, and these at a low frequency compared to the high rates of production. Differences would probably have been observed if the treatments had involved harvesting more leaves more often, and the observation period been longer. Our results are consistent with the life strategies described by [29], where palms in desert and xeric shrubland had more leaves in their crowns than palms in any other ecosystem. This would explain the plants’ lack of response to the treatments in our study.

During the 12 months of the study no mortality was observed. In other studies, there was no plant mortality reported for other species by [31, 32]. However, when evaluating the effects of leaf harvesting, it must be taken into account that effects are not limited to potential plant mortality but may also include changes in growth, such as decreased leaf length or decreased reproductive activity [33, 34]. Examining these aspects in *B. dulcis* could be subjects of future studies.

The insignificant effect that harvesting new and mature *B. dulcis* leaves has on individuals and populations is ideal from the perspective of NTFP exploitation, since harvesting of the resource does not appear to have significant implications, at least in the short term.

### Development of reproductive structures

*B. dulcis* has a biannual reproductive cycle, as do *Borassus aethiopum*[35] and *Oenocarpus bataua*[36]. Perhaps high leaf production rates in *B. dulcis* can be related to slow reproduction.

In spite of the reproductive activity of *B. dulcis* at the study sites, no recruitment of new individuals through sexual reproduction was observed during the study period. That is, no seeds were observed to germinate or seedlings to establish. This could be due to the high rate of solar radiation received at the site, trampling by livestock (sheep, goats and donkeys), and also partly because of seed depredation by weevils. In addition, there were cases of abortion, particularly in the transition from the flower to dry flower stage, and from dry flower to green fruit (Additional file 1). In the field, ants were often observed in the inflorescences and on the infructescences, and bees in the inflorescences, and fox feces containing seeds of this palm species were observed.

### Recommendations for sustainable use

1. As a first step, we propose apply in RBBM the traditional palm management practices used in Guerrero, including removing suckers and controlling grazing [15]. We consider it a mistake to invest money and effort in planting seeds at these locations (which is the simplistic option recommended by many managers), since this species regenerates easily if it is managed by proper sucker removal and controlled pasturing. Additional management might be required, but these two measures should be tried first, and the results evaluated.
2. Given that the few indigenous populations (Hñä hñü groups) living in the RBBM are the landholders and managers of these palm forests, and that the craftspeople are mostly women, the RBBM should promote the exploitation of this NTFP with a focus on ethnicity and gender, taking advantage of the need to promote sustainable activities in buffer zones.
3. Promotion of the use of any NTFP in PNA should take into account the lessons learned from comparative studies of NTFP around the world [37–39]. These studies show that a successful NTFP requires at the very least: a) assessing which NTFP have economic potential, and not naively assuming that the most biologically abundant NTFP has the greatest potential; b) understanding that in Latin America NTFPs are a complementary strategy to local economies; c) recognizing that although the key to success of an NTFP is in the economic sphere, biological limits and the maintenance of cultural values must be respected. In light of the above, it is recommended that RBBM officials design actions to: a) improve the quality of craftsmanship and product diversification; b) seek more and better fair trade markets for these products; c) provide a practical legal framework for the use of NTFP, rather than promoting conservation policies based on not managing resources.

## Conclusions

We suggest that *B. dulcis* is the most important palm with the highest potential for sustainable use in A–SA zones in Mexico because of its wide distribution across Mexico (13 states); the remarkable versatility of its parts, nearly all of which have a use; and the evident preponderance of this palm in the major traditional markets of Mexico. No other palm species in A-SA zones has as wide a geographic distribution or such wide use: for example other species of the genera *Brahea* and *Washingtonia* are typical of dry environments but are distributed in much smaller geographic areas, and are used only locally. For example, palmars (palm forests) composed of two *Brahea* and two *Washingtonia* species cover an area of 8533 ha in Baja California [40], while palmars of *B. dulcis* cover vast areas of unknown size in 13 states. Additionally, as was shown, the ecological behavior of *B. dulcis* includes the highest densities reported for the genus, and the highest rate of new leaf production per individual in spite of the arid conditions where it grows.

In spite of its huge potential, *B. dulcis* is not well managed in central Mexico because of legal framework, substitution and disuse. In the context of rural Mexico, it would be very complicated to comply with all legal requirements, as they set forth a set of procedures that would be difficult to follow. A reform of the official standards on palm harvesting in Mexico is therefore strongly recommended. Instead of requiring a series of bureaucratic procedures for obtaining the raw material, the procedures should be streamlined and simplified, and there should be an incentive to have agencies in place that could act as a communication bridge between rural residents and government agencies.

## Authors’ information

MTP: PhD, Science. Ethnobotany. Areas of research: non-timber forest products, population ecology and traditional farming systems. MCO: B.Sc. Biology. Areas of research: non-timber forest products.

## Electronic supplementary material

Additional file 1: **Details of fortnightly phenological stage of inflorescences (shaded) and infructescences of 18** ***B. dulcis*** **individuals followed for one year in the RBBM.** (DOC 348 KB)

## Abbreviations

A-SA: Arid and semi-arid areas
DL: Dry leaf
FL: First leaf
GL: Green leaf
GLM: Generalized linear model
NL: New leaf
NTFP: Non-timber forest product
PNA: Protected natural areas
RBBM: Barranca de Metztitlán biosphere reserves
s.e.: Standard error.

## References

[1] Johnson DV (2010). 10/Rev. 1 Tropical palms 2010 revision. Non-Wood Forest Products.

[2] Johnson DV, Wickens GE, Goodin JR, Field DV (1985). Present and Potential Economic Usages of Palms in Arid and Semi-Arid Areas. Proceedings of the Kew International Conference on Economic Plants for Arid Lands: 23–27 July 1984; Kew.

[3] Millennium Ecosystem Assessment (2005). Ecosystems and Human Well-Being: Desertification Synthesis.

[4] Aguilar J, Illsley C, Acosta J, Gómez T, Tlacotempa A, Flores A, Flores J, Miranda E, Sazoxoteco D, Teyuco E, López C, Chanfón S, Segura G (2005). Palma soyate. La riqueza de los bosques mexicanos: más allá de la madera. Experiencias de comunidades rurales.

[5] Blancas VJJ (2001). Estudio etnobotánico de soyatl o palma Brahea dulcis HBK Martius en la comunidad nahua de Huitziltepec.

[6] Illsley C, Aguilar J, Acosta J, García J, Gómez T, Caballero J, Rendon AB, Rebollar DS, Caballero J, Martínez M (2001). Contribuciones al conocimiento y manejo campesino de los palmares de *Brahea dulcis* (HKB) Mart. en la región de Chilapa, Guerrero. Plantas Cultura y Sociedad.

[7] Instituto Nacional de Investigaciones Forestales, Agrícolas y Pecuarias (INIFAP) (2008). Manual que establece los criterios técnicos para el aprovechamiento sustentable de recursos forestales no maderables de clima árido y semiárido. Subsecretaría de fomento y normatividad ambiental. Dirección general del sector primario y recursos naturales renovables.

[8] Mastache A, Morett E (1982). El trabajo de la palma en la región de La Montaña de Guerrero.

[9] Coronel-Ortega M (2010). Usos artesanales, fenología y cosecha óptima de la palma *Brahea dulcis* (Kunth) Mart. (Arecaceae), en dos zonas del Estado de Hidalgo. B Sc.

[10] Sánchez-Díaz G (1998). Los tejedores de palma. Manufacturas en Michoacán.

[11] Pavón N, Escobar R, Ortíz-Pulido R (2006). Extracción de hojas de la palma *Brahea dulcis* en una comunidad otomí en Hidalgo, México: efecto sobre algunos parámetros poblacionales. Interciencia.

[12] Ramírez-Rodríguez R, Tovar-Sánchez E, Jiménez-Ramírez J, Vega-Flores K, Rodríguez V (2011). Introgressive hybridization between *Brahea dulcis* and *Brahea nitida* (Arecaceae) en Mexico: evidence from morphological and PCR- RAPD patterns. Botany.

[13] Ramírez-Rodríguez R, Mussali-Galante P, Quero H, Tovar-Sánchez E (2012). Management and its relation to hybridization, clonality and genetic structure of the Mexican palm *Brahea dulcis*. Forest Ecol Manag.

[14] GEA (1996). Informe final del proyecto manejo campesino de recursos naturales de la selva baja caducifolia, en particular B. dulcis, en la región de Chilapa, Guerrero.

[15] Aguilar JG (1998). Manejo campesino de recursos naturales de la Selva Baja Caducifolia, en particular Brahea dulcis, en la región de Chilapa Guerrero: Segunda fase.

[16] Rzedowski J (2006). Vegetación de México.

[17] Quero H (2000). El complejo Brahea-Erythea (Palmae: Coryphoideae). Informe final* del Proyecto L216.

[18] Castillo GC (1993). Contribución al conocimiento sobre *Brahea dulcis* (H.B.K). Mart. en la región Mixteca de Cárdenas, Oaxaca. B Sc.

[19] Casas A, Caballero J, Mapes C, Zaráte S (1997). Manejo de la Vegetación, domesticación de plantas y origen de la agricultura en Mesoamérica. Bol Soc Bot Mex.

[20] CONANP Comisión Nacional de Áreas Naturales Protegidas (2003). Programa de manejo. Reserva de la Biosfera Barranca de Metztitlán.

[21] Balslev H, Navarrete H, Paniagua-Zambrana N, Pedersen D, Eiserhardt W, Kristiansen T (2010). El uso de transectos para el estudio de comunidades de palmas. Ecología en Bolivia.

[22] Haberman SJ (1973). The analysis of residuals in cross-classified tables. Biometrics.

[23] Pavón NP, Meza M (2009). Cambio climático en el estado de Hidalgo: clasificación de tendencias climáticas.

[24] Crawley MJ (1993). GLIM for Ecologist.

[25] Alexiades M, Shanley P (2004). Productos forestales, medios de subsistencia y conservación. Estudios de caso sobre sistemas de manejo de productos forestales no maderables.

[26] Pulido MT, Cuevas-Cardona C (2013). Cactus nurseries and conservation in a Biosphere Reserve in Mexico. Ethnobiol Lett.

[27] López-Toledo L, Horn C, Endress BA (2011). Distribution and population patterns of the threatened palm *Brahea aculeata* in a tropical dry forest in Sonora, Mexico. Forest Ecol Manag.

[28] González-Insuasti MS, Caballero J (2007). Managing plant resources: how intensive can it be?. Hum Ecol.

[29] Henderson A (2002). Evolution and Ecology of Palms.

[30] De Carvalho RM, Martins FR, Santos FAM (1999). Leaf Ecology of Pre-reproductive ontogenetic stages of the palm tree *Euterpe edulis* Mart. (Arecaceae). Ann Bot London.

[31] Martínez-Ballesté A, Martorell C, Caballero J (2008). The effect of Maya traditional harvesting on the leaf production, and demographic parameters of *Sabal* palm in the Yucatan Peninsula, Mexico. Forest Ecol Manag.

[32] Calvo-Irabien LM, Zapata MT, Iriarte-Vivar S (2009). Effects of leaf harvest on *Thrinax radiata* palm: implications for management and conservation. J Trop For Sci.

[33] Anten NPR, Martínez-Ramos M, Ackerly DD (2003). Defoliation and growth in a understory palm: quantifying the contributions of compensatory responses. Ecology.

[34] Endress B, Gorchov DL, Berry EJ (2006). Sustainability of a non-timber forest product: effects of alternative leaf harvest practices over 6 years on yield and demography of the palm *Chamaedorea radicalis*. Forest Ecol Manag.

[35] Barot S, Gignoux J (2007). Population structure and life cycle of *Borassus aethiopum* Mart. Evidence of early senescence in a palm tree. Biotropica.

[36] Miller C (2002). Fruit production of the ungurahua palm *Oenocarpus bataua* subsp. *bataua*, Arecaceae, in an indigenous Managed Reserve. Econ Bot.

[37] Marshall E, Schreckenberg K, Newton AC (2006). Comercialización de productos forestales no maderables, factores que influyen en el éxito. Conclusiones del estudio de México y Bolivia e implicancias políticas para los tomadores de decisiones.

[38] Pulido MT, González MS, Hersch P, Illsley C, López C, Ramírez F, Moreno A, Pulido MT, Mariaca R, Valadéz-Azúa R, Mejía Correa P, Gutiérrez-Santillán TV (2010). Productos forestales no maderables: consideraciones sobre su dimensión económica. Sistemas biocognitivos tradicionales: paradigmas en la conservación biológica y el fortalecimiento cultural.

[39] Ruiz-Pérez M, Belcher B, Achdiawan R, Alexiades M, Aubertin C, Caballero J, Campbell B, Clement C, Cunningham T, Fantini A, de Floresta H, García C, Gautam KH, Hersch P, de Jong W, Kusters K, Govindan M, López C, Fu M, Martínez-Alfaro MA, Raghavan TK, Ndoye O, Ocampo R, Rai N, Ricker M, Schreckenberg K, Schackleton S, Shanley P, Sunderland T, Youn Y (2004). Markets drive the specialization strategies of forest people. Ecol Soc.

[40] Minnich RA, Franco-Vizcaíno E, Salazar-Ceseña M (2011). Distribution and regional ecology of Californian palm oases interpreted from Google earth images. Aliso.

[41] Guarín JR, del Valle JI (2014). Modeling the stipe growth of the *Oenocarpus bataua* palm in the Central Cordillera of the Andes, Colombia. Forest Ecol Manag.

[42] Duarte N, Montúfar R (2012). Effect of leaf harvest on wax palm (*Ceroxylon echinalatum* Galeano) growth, and implications for sustainable management in Ecuador. Trop Conserv Sci.

[43] Velásquez Runk J (1998). Productivity and sustainability of a vegetable ivory palm (*Phytelephas aequatorialis*, Arecaceae) under three management regimes in Northwestern Ecuador. Econ Bot.

[44] Piñero D, Martínez-Ramos M, Sarukhán J (1984). A population model of *Astrocaryum mexicanum* and a sensitivity analysis of its finite rate of increase. J Ecol.

[45] Lugo A, Rivera Batlle CT (1987). Leaf production, growth rate, and age of the palm *Prestoea montana* in the Luquillo experimental forest, Puerto Rico. J Trop Ecol.

