# Supplementary material for: Ethnoecology of the palm Brahea dulcis (Kunth) Mart. in central Mexico
Source: J Ethnobiol Ethnomed. 2015 Jan 5;11:1. doi: 10.1186/1746-4269-11-1 (PMC4506432; doi:10.1186/1746-4269-11-1)
Supplement: Supplementary file 1 — Additional file 1: Details of fortnightly phenological stage of inflorescences (shaded) and infructescences of 18 B. dulcis individuals followed for one year in the RBBM. (DOC 348 KB) [file 13002_2014_466_MOESM1_ESM.doc]

**Appendix 1**

Details of fortnightly phenological stage of inflorescences (shaded) and infructescences of 18 *B. dulcis* individuals followed for one year in the RBBM. Inflorescences: e=emerging, p= pubescent, b=bud; f=flower; fs= dry flower. Infructescences: v= green, a=yellow, n=black, s=dry, x=abortive.

| (#) plant | Inflorescences/ Infructescences | **1** | **2** | **3** | **4** | **5** | **6** | **7** | **8** | **9** | **10** | **11** | **12** | **13** | **14** | **15** | **16** | **17** | **18** | **19** | **20** | **21** | **22** | **23** | **24** | **25** | **26** | **27** |
| --- | --- | --- | --- | --- | --- | --- | --- | --- | --- | --- | --- | --- | --- | --- | --- | --- | --- | --- | --- | --- | --- | --- | --- | --- | --- | --- | --- | --- |
| **10** | **1** | v | v | v | v | v | a | a | a | a | a | a | n | n | n | n | n | n | n | n | n | n | n | n | n | n | s | s |
| **10** | **2** | v | v | v | v | v | a | a | a | a | a | a | n | n | n | n | n | n | n | n | n | n | n | n | n | n | s | s |
| **10** | **3** | v | v | v | v | v | a | a | a | a | a | a | n | n | n | n | n | n | n | n | n | n | n | n | n | n | s | s |
| **10** | **4** | v | v | v | v | v | a | a | a | a | a | a | n | n | n | n | n | n | n | n | n | n | n | n | n | n | s | s |
| **10** | **5** | v | v | v | v | v | a | a | a | a | a | a | n | n | n | n | n | n | n | n | n | n | n | n | n | n | s | s |
| **10** | **6** | v | v | v | v | v | a | a | a | a | a | a | n | n | n | n | n | n | n | n | n | n | n | n | n | n | s | s |
| **10** | **7** | v | v | v | v | v | a | a | a | a | a | a | n | n | n | n | n | n | n | n | n | n | n | n | n | n | s | s |
| **11** | **1** | s | s | s | s | s | s | s | s | s | s | s | s | s | s | s | s | s | s | s | s | s | s | s | s | s | s | s |
| **11** | **2** | s | s | s | s | s | s | s | s | s | s | s | s | s | s | s | s | s | s | s | s | s | s | s | s | s | s | s |
| **11** | **3** | s | s | s | s | s | s | s | s | s | s | s | s | s | s | s | s | s | s | s | s | s | s | s | s | s | s | s |
| **11** | **4** | s | s | s | s | s | s | s | s | s | s | s | s | s | s | s | s | s | s | s | s | s |  |  |  |  |  |  |
| **11** | **5** | s | s | s | s | s | s | s | s | s | s | s | s | s | s | s | s | s | s | s | s |  |  |  |  |  |  |  |
| **11** | **6** | s | s | s | s | s | s | s | s | s | s | s | s | s | s | s | s |  |  |  |  |  |  |  |  |  |  |  |
| **11** | **7** | s | s | s | s |  |  |  |  |  |  |  |  |  |  |  |  |  |  |  |  |  |  |  |  |  |  |  |
| **11** | **8** |  |  |  |  |  |  |  |  |  |  |  |  |  |  |  |  |  |  |  |  | e | e | p | p | p | p | p |
| **11** | **9** |  |  |  |  |  |  |  |  |  |  |  |  |  |  |  |  |  |  |  |  | e | e | e | p | p | p | p |
| **11** | **10** |  |  |  |  |  |  |  |  |  |  |  |  |  |  |  |  |  |  |  |  | e | e | e | p | p | p | p |
| **11** | **11** |  |  |  |  |  |  |  |  |  |  |  |  |  |  |  |  |  |  |  |  | e | e | e | p | p | p | p |
| **11** | **12** |  |  |  |  |  |  |  |  |  |  |  |  |  |  |  |  |  |  |  |  | e | e | e | p | p | p | p |
| **13** | **1** | b | b | b | b | b | b | b | b | b | b | b | b | f | f | f | f | f | fs | fs | fs | v | v | v | v | v | v | v |
| **16** | **1** | s | s | s | s | s | s | s | s | s | s | s | s | s | s | s | s | s | s | s | s |  |  |  |  |  |  |  |
|  | **2** | s | s |  |  |  |  |  |  |  |  |  |  |  |  |  |  |  |  |  |  |  |  |  |  |  |  |  |
|  | **3** |  |  |  |  |  |  |  |  |  |  |  |  |  |  |  |  |  |  |  | e | e | e | p | p | p | p | p |
|  | **4** |  |  |  |  |  |  |  |  |  |  |  |  |  |  |  |  |  |  |  | e | e | e | p | p | p | p | p |
|  | **5** |  |  |  |  |  |  |  |  |  |  |  |  |  |  |  |  |  |  |  | e | e | e | p | p | p | p | p |
|  | **6** |  |  |  |  |  |  |  |  |  |  |  |  |  |  |  |  |  |  |  | e | e | e | p | p | p | p | p |
|  | **7** |  |  |  |  |  |  |  |  |  |  |  |  |  |  |  |  |  |  |  | e | e | e | p | p | p | p | p |
|  | **8** |  |  |  |  |  |  |  |  |  |  |  |  |  |  |  |  |  |  |  |  | e | e | e | p | p | p | p |
| **17** | **1** | b | b | b | b | b | b | b | b | b | f | f | f | f | f | fs | fs | fs | fs | fs | x | x | x | x | x | x | x | x |
|  | **2** | b | b | b | b | b | b | b | b | f | f | f | f | f | f | fs | fs | fs | fs | fs | x | x | x | x | x | x | x | x |
|  | **3** | b | b | b | b | b | b | b | b | f | f | f | f | f | f | fs | fs | fs | fs | fs | x | x | x | x | x | x | x | x |
|  | **4** | b | b | b | b | b | b | b | f | f | f | f | f | f | f | fs | x | x | x | x | x | x | x | x | x | x | x | x |
|  | **5** | b | b | b | b | b | b | b | f | x | x | x | x | x | x | x | x | x | x | x | x | x | x | x | x | x | x | x |
| **19** | **1** | b | b | b | b | b | b | b | b | b | b | b | b | b | b | b | b | f | f | f | f | x | x | x | x | x | x | x |
|  | **2** | b | b | b | b | b | b | b | b | b | b | b | b | b | b | b | b | x | x | x | x | x | x | x | x | x | x | x |
|  | **3** | b | b | b | b | b | b | b | b | b | b | b | b | b | b | b | b | x | x | x | x | x | x | x | x | x | x | x |
|  | **4** | v | v | v | v | v | a | a | a | n | n | n | n | s | s | s | s | s | s | s | s | s | s | s | s | s | s | s |
| **20** | **1** | b | b | b | b | b | b | b | b | b | b | f | f | f | f | f | f | f | fs | fs | fs | v | v | v | v | v | v | v |
|  | **2** | v | v | v | v | v | a | a | a | a | a | a | a | a | a | a | n | n | n | n | n | n | n | n | s | s | s | s |
|  | **3** | v | v | v | v | v | a | a | a | a | a | a | a | a | a | a | n | n | n | n | n | n | n | n | s | s | s | s |
|  | **4** | v | v | v | v | v | a | a | a | a | a | a | a | a | a | a | n | n | n | n | s | s | s | s | s | s | s | s |
|  | **5** | v | v | v | v | v | a | a | a | a | a | a | s | s | s | s | s | s | s | s | s | s | s | s | s | s | s | s |
| **21** | **1** |  |  |  |  |  |  |  |  |  |  |  |  |  |  |  |  |  |  |  |  | e | e | e | p | p | p | p |
| **23** | **1** | b | b | b | b | b | b | b | b | b | b | b | f | f | f | f | f | f | f | f | f | f | f | fs | fs | v | v | v |
|  | **2** | b | b | b | b | b | b | b | b | b | b | b | b |  | f | f | f | f | f | f | f | f | f | f | f | f | f | f |
|  | **3** | b | b | b | b | b | b | b | b | b | b | b | b | b | f | f | f | f | f | f | f | f | f | f | f | f | f | f |
|  | **4** | p | p | p | p | p | p | p | p | p | p | p | p | p | p | b | b | b | b | b | b | f | f | f | f | f | f | f |
|  | **5** | p | p | p | p | p | p | p | p | p | p | p | p | p | p | b | b | b | b | b | f | f | f | f | f | x | x | x |
|  | **6** | p | p | p | p | p | p | p | p | p | p | p | p | p | p | b | b | b | b | b | f | f | f | f | f | x | x | x |
|  | **7** | v | v | v | v | v | a | a | a | a | a | n | n | n | n | s | s | s | s | s | s | s | s | s | s | s | s | s |
|  | **8** | s | s | s | s | s | s | s | s | s | s | s | s | s | s | s | s | s | s | s | s | s | s | s | s | s | s | s |
|  | **9** | s | s | s | s | s | s | s | s | s | s | s | s | s | s | s | s | s | s | s | s | s | s | s | s | s | s | s |
|  | **10** | s | s | s | s | s | s | s | s | s | s | s | s | s | s | s | s | s | s | s | s | s | s | s | s | s | s | s |
|  | **11** | s | s | s | s | s | s | s | s | s | s | s | s | s | s | s | s | s | s | s | s | s | s | s | s | s | s | s |
|  | **12** | s | s | s | s | s | s | s | s | s | s | s | s | s | s | s | s | s | s | s | s | s | s | s | s |  |  |  |
|  | **13** | s | s | s | s | s | s | s | s | s | s | s | s | s | s | s | s | s | s | s | s |  |  |  |  |  |  |  |
|  | **14** | s | s | s | s | s | s | s | s | s | s | s | s | s | s | s | s | s | s | s | s |  |  |  |  |  |  |  |
| **25** | **1** | b | b | b | b | b | b | b | b | b | b | f | f | f | f | f | f | f | f | f | f | fs | fs | v | v | v | v | v |
|  | **2** | v | v | v | v | v | v | v | v | v | v | v | v | v | v | v | a | a | a | a | n | n | n | n | n | s | s | s |
|  | **3** | v | v | v | v | v | v | v | v | v | v | v | v | v | v | v | a | a | a | a | n | n | n | n | n | s | s | s |
|  | **4** | v | v | v | v | v | v | v | v | v | v | v | v | v | v | v | a | a | a | n | n | n | n | n | n | s | s | s |
|  | **5** | v | v | v | v | v | v | v | v | v | v | v | v | v | v | v | a | n | n | n | n | n | n | s | s | s | s | s |
| **26** | **1** | v | v | v | v | v | v | v | v | v | v | v | v | v | v | v | v | a | a | a | n | n | n | n | n | s | s | s |
|  | **2** | v | v | v | v | v | v | v | v | v | v | v | v | v | v | v | v | a | a | a | n | n | n | n | n | s | s | s |
|  | **3** | v | v | v | v | v | v | v | v | v | v | v | v | v | v | a | a | a | a | n | n | n | n | n | n | s | s | s |
|  | **4** | v | v | v | v | v | v | v | v | v | v | v | v | v | v | a | a | a | a | n | n | n | n | s | s | s | s | s |
|  | **5** | v | v | v | v | v | v | v | v | v | v | v | v | v | v | a | a | a | a | n | n | n | s | s | s | s | s | s |
| **27** | **1** | v | v | v | v | v | v | v | v | v | v | v | v | v | v | a | n | n | n | n | n | n | n | s | s | s | s | s |
| **30** | **1** | b | b | b | b | b | f | f | f | fs | v | s | s | s | s | s | s | s | s | s | s | s | s | s | s | s | s |  |
| **34** | **1** | v | v | v | v | v | v | v | v | v | v | v | v | v | v | v | v | v | a | a | n | n | n | n | s | s | s | s |
|  | **2** | v | v | v | v | v | v | v | v | v | v | v | v | v | v | v | v | v | a | a | n | n | n | n | s | s | s | s |
|  | **3** | v | v | v | v | v | a | a | a | s | s | s | s | s | s | s | s | s | s | s | s | s | s | s | s | s | s | s |
|  | **4** | v | v | v | v | v | a | a | a | s | s | s | s | s | s | s | s | s | s | s | s | s | s | s | s | s | s | s |
|  | **5** | v | v | v | v | v | a | a | a | s | s | s | s | s | s | s | s | s | s | s | s | s | s | s | s | s | s | s |
|  | **6** | v | v | v | v | v | a | a | a | s | s | s | s | s | s | s | s | s | s | s | s | s | s | s | s | s | s | s |
|  | **7** | v | v | v | v | v | a | a | s | s | s | s | s | s | s | s | s | s | s | s | s | s | s | s | s | s | s | s |
| **38** | **1** | b | b | b | b | b | b | f | f | f | f | fs | fs | fs | fs | fs | fs | fs | fs | fs | fs | fs | fs | x | x | x | x | x |
|  | **2** | b | b | b | f | f | fs | fs | fs | fs | fs | fs | fs | fs | fs | fs | fs | fs | fs | fs | fs | fs | fs | x | x | x | x | x |
|  | **3** | b | b | b | f | f | fs | fs | fs | fs | fs | fs | fs | fs | fs | fs | fs | fs | fs | fs | fs | fs | fs | x | x | x | x | x |
|  | **4** | b | b | b | f | f | fs | fs | fs | fs | fs | fs | fs | fs | fs | fs | fs | fs | fs | fs | fs | fs | fs | x | x | x | x | x |
|  | **5** | s | s | s | s | s | s | s | s | s | s | s | s | s | s | s | s | s | s | s | s | s | s | s | s | s | s | s |
|  | **6** | s | s | s | s | s | s | s | s | s | s | s | s | s | s | s | s | s | s | s | s |  |  |  |  |  |  |  |
|  | **7** | s | s | s | s | s | s | s | s | s | s | s | s | s | s | s |  |  |  |  |  |  |  |  |  |  |  |  |
| **39** | **1** | v | a | a | a | a | a | a | a | a | a | n | n | n | n | n | n | n | s | s | s | s | s | s | s | s | s | s |
|  | **2** | v | a | a | a | a | a | a | a | a | a | n | n | n | n | n | n | n | s | s | s | s | s | s | s | s | s | s |
|  | **3** | v | a | a | a | a | a | a | a | a | a | n | n | n | n | n | n | s | s | s | s | s | s | s | s | s | s | s |
|  | **4** | v | a | a | a | a | a | a | a | a | a | n | n | n | n | n | n | s | s | s | s | s | s | s | s | s | s | s |
|  | **5** |  |  |  |  |  |  |  |  |  |  |  |  |  |  |  |  |  |  |  | e | e | e | p | p | p | p | p |
|  | **6** |  |  |  |  |  |  |  |  |  |  |  |  |  |  |  |  |  |  |  | e | e | e | p | p | p | p | p |
| **41** | **1** | b | b | b | b | b | b | b | b | b | b | b | b | b | b | b | b | f | x | x | x | x | x | x | x | x | x | x |
| **42** | **1** | b | b | b | b | b | b | b | b | b | b | b | b | b | b | b | b | b | f | f | f | f | x | x | x | x | x | x |
|  | **2** | b | b | b | b | b | b | b | b | b | b | b | b | b | b | b | b | b | f | f | f | x | x | x | x | x | x | x |
|  | **3** | s | s | s | s | s | s | s | s | s | s | s | s | s | s | s | s | s | s | s | s | s | s | s | s | s | s | s |
|  | **4** | s | s | s | s | s | s | s | s | s | s | s | s | s | s | s | s | s | s | s | s | s | s | s | s | s | s |  |
